# The Outcomes of Transabdominal Preperitoneal Repair for Groin Hernias In a Single Consultant Series

**DOI:** 10.7759/cureus.92376

**Published:** 2025-09-15

**Authors:** Sadaf Khalid, Mena Alwhouhayb, Rimsha Rana, Gaurav Maheshwari, Ahmed Elmoraly, Luis Soares

**Affiliations:** 1 General Surgery, Royal Free NHS Foundation Trust, London, GBR; 2 General Surgery, Royal Free NHS Foundation Trust Barnet Hospital, London, GBR; 3 General and Colorectal Surgery, Medway Maritime NHS Foundation Trust, Kent, GBR; 4 General Surgery, East Sussex Healthcare NHS Trust, Hastings, GBR; 5 General Surgery, Barnet Hospital Royal Free NHS Foundation Trust, London, GBR

**Keywords:** chronic postoperative pain, inguinal hernia, laparoscopic tapp repair, surgical outcomes, synthetic mesh

## Abstract

Background

Inguinal hernia repair is one of the most commonly performed operations in general surgery. The laparoscopic transabdominal preperitoneal approach gives a better view of the inguinal anatomy, and the procedure also has a short learning curve. We aim to evaluate the safety and outcomes of laparoscopic transabdominal preperitoneal (TAPP) repair using the synthetic mesh in a single consultant series and compare the outcomes with the available literature.

Methods

Retrospective evaluation of the outcomes was carried out on all patients who underwent laparoscopic TAPP repair from September 2018 to June 2024 by analyzing the postoperative morbidity, which includes postoperative complications, seroma, hematoma, wound infection, recurrence, post op chronic pain persisting beyond three months, and representation. Data was analyzed from the electronic patient records (EPR) and assessment of the chronic pain via assessing the available clinic follow-ups on EPR and the telephonic interviews using the European Registry for the Abdominal Wall Hernias of Life (EuraHS-Qol) questionnaire.

Results

A total of 140 patients underwent elective TAPP repairs. One hundred and eight (77.1%) were male and 32 (22.8%) were female. The median age was 54 years (range 22-89). Forty-one (29.2%) patients underwent bilateral repair, and three patients were found to have a femoral hernia. None of the cases required conversion to open repair. Seventy-eight (55.7%) patients were successfully contacted for a telephonic interview (mean duration six to eight minutes) to assess chronic pain and its impact on daily activity. Twelve (8.57%) patients reported some degree of discomfort, of whom eight (5.7%) reported chronic pain at rest with exacerbation on activity. Eighteen (12.9%) patients had postoperative complications (seroma, scrotal swelling, wound infection). Three patients required clinic or hot-clinic follow-up for hematomas. Median length of stay was one day. Readmission rate for postoperative scrotal swelling secondary to seroma was 9.4%. Two (1.42%) patients had recurrence at 12-14 months and subsequently underwent open repair.

Conclusion

The primary outcomes of chronic postoperative pain, recurrence rate, and postoperative morbidity were comparable to the available literature. There was no conversion to open in our study. However, there were no major complications, making TAPP repair a safe choice for day case surgery.

## Introduction

An inguinal hernia is a major cause of small intestinal obstruction and is termed "incarcerated" when it cannot be manually reduced back into the abdominal cavity [1]. In elderly and frail individuals, particularly women, incarcerated inguinal hernias carry a mortality rate of up to 5% [2]. Inguinal hernia is commonly defined as the protrusion of intra-abdominal fat or bowel through a weakness in the inguinal canal [3]. The incidence of inguinal hernias demonstrates a bimodal distribution, with one peak in childhood and a second in older age groups, especially in men. Since our study population only included adults (≥21 years), the pediatric peak is not relevant to this series, and the focus is on the adult incidence. The lifetime risk of undergoing an inguinal hernia repair is considerably high, estimated at 27% for men and 3% for women [4-6].

Due to the risk of complications, inguinal hernias should ideally be treated surgically, even if asymptomatic. Inguinal hernia repair is one of the most frequently performed surgeries worldwide and represents a considerable economic burden on healthcare systems [3,7]. The primary indicators of successful groin hernia surgery include minimal complication rates, favorable cost-effectiveness compared to open repair, durable repair, and early return to normal activity. Among these, recurrence remains a critical determinant of surgical success. Prior to the widespread adoption of mesh-based techniques, recurrence rates following primary tissue repairs were reported to exceed 15% [8].

With the advent and increasing utilization of nonabsorbable mesh in both open and laparoscopic approaches, recurrence rates have significantly declined. However, recurrences continue to occur due to various factors. Repairing recurrent inguinal hernias poses a greater technical challenge for surgeons, as previous surgical scarring can alter anatomy and thin tissue layers, increasing the risk of further recurrence or complications [9].

Laparoscopic inguinal hernia repair, particularly the transabdominal preperitoneal (TAPP) approach, has gained popularity due to its association with reduced postoperative pain, lower incidence of wound-related complications, early return to activity, and decreased chronic pain [10]. The TAPP technique is widely recognized as a safe and effective option for both primary and recurrent groin hernia repairs [11,12]. Furthermore, it allows for the evaluation of the peritoneal cavity and the detection of bilateral hernias without requiring additional dissection [13]. Despite these advantages, there is still limited evidence supporting laparoscopic approaches, including TAPP, in emergency presentations. In such cases, bowel viability must be confirmed, often necessitating diagnostic laparoscopy as part of the management strategy. There is a lack of localized, single-surgeon data evaluating the outcomes of TAPP repair, particularly in terms of recurrence, complications, and postoperative quality of life. The objective of this study is to assess the safety, feasibility, and outcomes of laparoscopic TAPP repair using synthetic mesh for groin hernias in a single consultant series, and to compare these findings with existing literature.

## Materials and methods

This retrospective observational study was conducted at the Department of General Surgery, Barnet Hospital, Royal Free NHS Foundation Trust, a tertiary care center in London, United Kingdom. The study evaluated the clinical outcomes of patients who underwent laparoscopic transabdominal preperitoneal (TAPP) repair for groin hernias under the care of a single consultant surgeon between September 2018 and June 2024.

The study included all adult patients (aged ≥18 years) who underwent elective laparoscopic TAPP repair for primary or recurrent unilateral or bilateral groin hernias during the defined study period. The sample size was calculated using the OpenEpi calculator, taking an expected postoperative complication rate of 10%, a 95% confidence level, and a 5% margin of error. Based on this calculation, the minimum required sample size was 140 patients.

Non-probability consecutive sampling was employed, including all patients meeting the inclusion criteria who underwent surgery during the study period by the designated consultant. Inclusion criteria were adult patients undergoing laparoscopic TAPP repair for direct, indirect, or combined inguinal hernias, with complete postoperative follow-up records available in the EPR system. Patients were excluded if they had incomplete data, underwent emergency hernia repair, had concurrent abdominal surgical procedures, were lost to follow-up, or declined participation in telephonic follow-up interviews.

Data collection was performed by accessing the EPR system to extract demographic details (age, gender, BMI), comorbidities, hernia characteristics (type, laterality, primary/recurrent), operative details (operative time, mesh fixation technique), and postoperative outcomes. The primary outcomes assessed were postoperative morbidity and recurrence. Postoperative complications included seroma, hematoma, wound infection, and chronic groin pain lasting beyond three months. Data on early postoperative complications (within 30 days of surgery) were collected from inpatient and outpatient EPR documentation. Assessment of chronic pain and quality of life was done through available clinic follow-up records and supplemented by structured telephonic interviews using the standardized European Registry for Abdominal Wall Hernias Quality of Life (EuraHS-QoL) questionnaire [14]. The EuraHS-QoL questionnaire was used in this academic, non-commercial study with acknowledgment to the European Hernia Society. The tool is freely available for academic and clinical research, and was originally developed and validated previously [14].

Recurrence was identified through clinical records, radiological findings where available, and patient-reported outcomes during follow-up. Pain scores were categorized as mild, moderate, or severe based on EuraHS-QoL responses. In cases where follow-up clinic visits were missing, patients were contacted via telephone, and verbal consent was obtained before administering the questionnaire.

All collected data were anonymized and analyzed using SPSS Statistics version 26 (IBM Inc., Armonk, New York). Continuous variables, including age, BMI, and operative time, were assumed to be normally distributed and presented as mean ± standard deviation (SD). Categorical variables such as type of hernia, occurrence of complications, and recurrence rates were expressed as frequencies and percentages. For comparison of means between groups (e.g., those with and without complications), the independent samples t-test was applied. Associations between categorical variables were analyzed using the Chi-squared test. A p-value of less than 0.05 was considered statistically significant.

## Results

A total of 140 patients who underwent elective laparoscopic transabdominal preperitoneal (TAPP) groin hernia repair between September 2018 and June 2024 by a single consultant surgeon were included in the final analysis. The baseline demographic and clinical characteristics are summarized in Table 1. The majority of patients were male (110/140, 77.1%), with a mean age of 54.3 ± 15.7 years (range: 22-89 years). The mean BMI was 26.4 ± 3.8 kg/m². Hypertension was the most common comorbidity (29.2%), followed by diabetes mellitus (17.1%) and smoking (15.7%).

Of the 140 patients, 41 (29.2%) underwent bilateral repair. Most hernias were inguinal; however, three patients (2.1%) had femoral hernias. All procedures were completed laparoscopically with no conversions to open surgery. The median length of hospital stay was one day (range one to three days; Table 1).

**Table 1:** Baseline demographic and clinical characteristics of patients (n=140) Baseline data including patient age, gender distribution, BMI, comorbidities (hypertension, diabetes, smoking), hernia laterality and type, history of previous hernia surgery, conversion to open surgery, and length of hospital stay.

Parameter	Value
Mean age (years)	54.3 ± 15.7
Gender (male/female)	110 (77.1%) / 32 (22.8%)
BMI (kg/m²)	26.4 ± 3.8
Comorbidities	
Hypertension	41 (29.2%)
Diabetes mellitus	24 (17.1%)
Smoker	22 (15.7%)
Hernia laterality	
Unilateral	99 (70.7%)
Bilateral	41 (29.2%)
Hernia type	
Inguinal	137 (97.8%)
Femoral	3 (2.1%)
Previous hernia surgery	19 (13.5%)
Conversion to open surgery	0
Median length of stay (days)	1 (range: 1–3)

Operative outcomes and postoperative complications

The mean operative time for unilateral repair was 46.5 ± 9.2 minutes, while bilateral repairs averaged 68.7 ± 11.4 minutes. A total of 18 patients (12.8%) experienced early postoperative complications. The most common complication was scrotal swelling (7.8%), followed by seroma formation (3.5%) and wound infection (1.4%). Three patients (2.1%) presented with postoperative hematomas during follow-up visits. The overall readmission rate due to complications, primarily scrotal swelling and symptomatic seromas, was 9.4%. No mesh infections were reported (Table 2).

**Table 2:** Operative details and early postoperative complications Operative time for unilateral and bilateral hernia repairs, frequency and type of early postoperative complications, readmission rate, and incidence of conversion to open repair.

Parameter	Value
Mean operative time (unilateral)	46.5 ± 9.2 minutes
Mean operative time (bilateral)	68.7 ± 11.4 minutes
Early postoperative complications	18 (12.8%)
Seroma	5 (3.5%)
Scrotal Swelling	11 (7.8%)
Wound Infection	2 (1.4%)
Hematoma (clinic follow-up)	3 (2.1%)
Readmission rate	13 (9.4%)
Conversion to open repair	0

Chronic pain and quality of life outcomes

All 140 patients were contacted for telephonic follow-up using the EuraHS-QoL questionnaire at a median of 12 months postoperatively. The average duration of each interview was six to eight minutes. Chronic pain was defined as pain persisting beyond three months after surgery.

Among the 140 patients, 12 (8.6%) reported some form of chronic discomfort or pain. Eight (5.7%) specifically reported chronic groin pain persisting at rest, which was exacerbated by physical activity, while four (2.9%) reported only mild activity-related discomfort without pain at rest. Most of these patients had bilateral repairs or associated early postoperative complications, which were analyzed as potential confounders.

Pain severity scores (0-10) from the EuraHS-QoL indicated a mean score of 1.4 ± 0.8 among those with chronic pain. No patients reported severe limitations in daily activities or required long-term analgesics. Chronic pain was more frequently observed in patients with prior hernia repair or those who had scrotal swelling or seroma postoperatively (p=0.03, independent samples t-test; see Table 3).

**Table 3:** Chronic pain and quality of life assessment (n=78) Assessment of chronic pain and discomfort among followed-up patients, including pain at rest, activity-related pain, pain scores, impact on daily activities, duration of follow-up interviews, and association with early complications.

Parameter	Value
Average duration of interview (minutes)	6–8
Any chronic pain/discomfort	12 (8.57%)
Chronic pain at rest	8 (5.7%)
Activity-induced pain without rest pain	4 (2.8%)
Mean Pain Score (0–10)	1.4 ± 0.8
Impact on daily activities (moderate-severe)	0
Patients with early complications and pain	9/18 (50.0%)
Statistical association (p-value)	p = 0.03

Hernia recurrence

Two patients (2/140; 1.42%) experienced hernia recurrence within 12-14 months postoperatively. Both cases involved patients with bilateral hernias and a prior surgical history, and were subsequently managed with open mesh repair. These patients had presented with persistent discomfort during follow-up and were identified through clinic records as well as telephonic reporting (Table 4).

**Table 4:** Hernia recurrence characteristics Details of patients with hernia recurrence, including number of recurrences, time to recurrence, location, prior hernia repair history, surgical management, and association with early postoperative complications.

Parameter	Value
Total recurrences	2 (1.42%)
Time to recurrence	12–14 months
Location	Bilateral in both cases
Prior hernia repair	Yes (both cases)
Surgical Management	Open mesh repair
Association withearly complication	Yes (1 out of 2 cases)

Association of comorbidities with postoperative complications

To evaluate the influence of patient comorbidities on the incidence of postoperative complications, a comparative analysis was conducted using the independent samples t-test for continuous variables and the Chi-squared test for categorical data. Patients with hypertension, diabetes, and a smoking history were observed to have a higher frequency of postoperative complications compared to those without comorbidities. The association was statistically significant for hypertension (p=0.02) and diabetes mellitus (p=0.04), but not for smoking (p=0.09; Table 5). Patients with both hypertension and diabetes had a compounded risk, especially for seroma formation and scrotal swelling. These findings suggest the importance of optimizing comorbid conditions before elective TAPP repair.

**Table 5:** Association of comorbidities with postoperative complications Distribution of postoperative complications among patients with and without comorbidities (hypertension, diabetes, smoking), along with p-values indicating statistical association.

Comorbidity	Patients with comorbidity (n)	Patients with complications (n/%)	p-value
Hypertension	41	9 (21.9%)	0.02
Diabetes mellitus	24	6 (25.0%)	0.04
Smoking	22	3 (13.6%)	0.09
No comorbidity	53	3 (5.7%)	—

EuraHS-QoL outcomes: pain, activity, and cosmetic domains

Among the 78 patients who were successfully contacted and completed the EuraHS-QoL telephonic interview, responses were recorded in three domains: pain, activity limitation, and cosmetic discomfort, each rated on a 0-10 scale. Patients with comorbidities reported higher mean scores in pain and activity domains, indicating reduced quality of life postoperatively. Cosmetic satisfaction was generally high across all groups (Table 6).

**Table 6:** EuraHS-QoL outcomes in pain, activity, and cosmetic domains (n=78) EuraHS-QoL scores for pain, activity limitation, and cosmetic discomfort, stratified by presence or absence of comorbidities, including mean ± SD and associated p-values.

Parameter	All patients (mean ± SD)	With comorbidity (n = 43)	Without comorbidity (n = 35)	p-value
Pain score (0–10)	1.4 ± 0.8	1.9 ± 1.1	0.9 ± 0.5	0.01
Activity limitation (0–10)	1.2 ± 0.6	1.6 ± 0.9	0.8 ± 0.4	0.02
Cosmetic discomfort (0–10)	0.6 ± 0.4	0.7 ± 0.5	0.5 ± 0.3	0.21

Patients with comorbidities-especially diabetes and hypertension-reported significantly higher pain and activity limitation scores (p=0.01 and p=0.02, respectively). However, the cosmetic satisfaction domain showed no statistically significant difference (p=0.21), indicating that visual outcomes remained unaffected by systemic health status.

Comorbidity status was a notable confounder affecting both complication rates and quality-of-life outcomes. Multivariate adjustment for age, gender, bilateral repair, and previous hernia repair further strengthened the independent association of hypertension and diabetes with adverse outcomes. Stratified analysis showed that older patients (>60 years) with comorbidities had the highest complication burden and the lowest QoL scores.

## Discussion

This study aimed to evaluate the outcomes of transabdominal preperitoneal (TAPP) repair for groin hernias performed by a single consultant, focusing on demographic characteristics, intraoperative parameters, postoperative complications, chronic pain, quality of life, and recurrence. The findings from our series of 140 patients align well with existing literature while offering some distinctive insights due to the uniformity of surgical technique across all cases.

The mean age of patients in our study was 54.3 years, with a male predominance (77.1%), which is consistent with global epidemiological data reporting that inguinal hernias are significantly more common in men, particularly in older age groups [15, 16]. Our cohort included 29.2% bilateral hernias and 13.5% with a history of previous hernia surgery, reflecting typical presentation patterns reported in similar studies [17]. The observed mean BMI of 26.4 kg/m² places most patients in the overweight category, which has been identified in prior studies as a potential contributor to increased intra-abdominal pressure and hernia formation, though its exact role in recurrence and complication rates remains debated [18].

The mean operative time in our study was 46.5 minutes for unilateral and 68.7 minutes for bilateral hernias, which is shorter than the operative durations reported by Hidalgo et al. [19] and Krishna et al. [20], who noted average times exceeding 70 minutes, especially for bilateral repairs. This discrepancy could be attributed to surgical proficiency, as our series was performed by a single experienced consultant, thereby reducing variability.

The overall complication rate of 12.8% in our cohort was within the range reported in recent literature. Scrotal swelling (7.8%) and seroma formation (3.5%) were the most common issues, followed by hematoma (2.1%) and wound infection (1.4%). Our findings align with the work of Abdelsamie et al. [21], who reported a 9% incidence of seroma in TAPP repairs, and Scheuermann et al. [12], who found hematoma and wound infections to be relatively rare complications. The low infection rate further supports the safety of mesh use in clean and clean-contaminated fields, as endorsed by current guidelines [22, 23].

The readmission rate in our study (9.4%), primarily due to symptomatic seromas, is comparable to that reported in previous observational studies, suggesting that most complications following TAPP are minor and manageable without reoperation [24].

Chronic postoperative pain was reported by 8.57% of patients, a figure that falls on the lower end of the spectrum compared to previous studies, where chronic pain rates ranged from 10% to as high as 16% [25]. Pain at rest was reported by 5.7%, while activity-induced pain was observed in 2.8%. These results are consistent with those of Krishna et al.'s use of the EuraHS-QoL tool in our study, which added robustness to pain and QoL assessment, as it is validated and concise, as highlighted by Molegraaf et al. [26] and originally developed by Muysoms et al. [14].

Our analysis revealed a statistically significant association between early postoperative complications and the development of chronic pain (p=0.03), which has been supported in prior research as early inflammation and tissue trauma may predispose to persistent discomfort [26, 27]. Comorbidities such as hypertension and diabetes also demonstrated a significant association with higher pain and activity limitation scores (p=0.01 and 0.02, respectively), reaffirming findings by Kim et al. [28] and Krishna et al. [20], who suggested systemic conditions may exacerbate postoperative sensory symptoms.

Cosmetic dissatisfaction scores remained low overall (mean 0.6), with no significant differences between patients with or without comorbidities. This underscores one of the advantages of laparoscopic repair - superior aesthetic outcomes due to minimal incisions - consistent with conclusions from multiple comparative trials [29].

The recurrence rate in our study was 1.42%, comparable to or better than recurrence rates reported in other TAPP studies, which range from 1% to 6% [12,20,30]. Notably, both recurrences in our cohort occurred in patients with prior repairs and bilateral hernias, factors repeatedly identified in the literature as predictors of recurrence due to anatomical distortion and scar tissue [30]. No cases required immediate reoperation, and both recurrences were managed with open mesh repair. The low recurrence rate demonstrates that TAPP repair, when performed by experienced hands, offers durable outcomes even in complex bilateral or recurrent cases.

Patients with comorbidities, particularly hypertension and diabetes, were found to have significantly higher rates of postoperative complications (p=0.02 and 0.04, respectively). These comorbidities are known to affect wound healing, increase infection risk, and may contribute to seroma formation due to vascular fragility and impaired lymphatic drainage [21, 22]. This reinforces the importance of preoperative optimization in patients with chronic conditions undergoing TAPP repair.

Our findings confirm the growing body of evidence supporting laparoscopic TAPP repair as a safe and effective approach for elective groin hernia surgery. Consistent with the results of multiple studies, including those by Wilson et al. [23], Bittner et al. [24], and Hidalgo et al. [19], our study demonstrated lower rates of pain, infection, and recurrence compared to traditional open repair techniques. Unlike the TEP approach, which is technically more demanding and associated with a steeper learning curve, TAPP allows for thorough inspection of the abdominal cavity and detection of occult contralateral hernias-an added advantage emphasized in several reviews [20].

One of the strengths of this study is the uniformity of surgical technique, as all procedures were performed by a single consultant, reducing variability. Additionally, the use of a validated QoL tool (EuraHS-QoL) enhances the reliability of patient-reported outcomes. However, the retrospective design and relatively modest sample size, along with partial loss to follow-up (only 78 of 140 patients completed the QoL assessment), are important limitations. Moreover, while the recurrence and complication rates are low, longer-term follow-up may reveal additional outcomes not captured within the current timeframe.

## Conclusions

The findings support the safety and efficacy of laparoscopic TAPP repair in elective groin hernia surgery. The procedure demonstrates low complication and recurrence rates, high patient satisfaction, and acceptable levels of chronic pain. The presence of comorbidities was associated with higher complication rates and poorer QoL outcomes, emphasizing the need for careful patient selection and optimization. These results reaffirm existing literature and strengthen the case for TAPP repair as a standard technique in the elective management of groin hernias. Further prospective studies with larger cohorts and extended follow-up are warranted to validate these findings and explore strategies to further minimize chronic pain and recurrence.
